# Diagnosis and Management of Marchiafava-Bignami Disease, a Rare Neurological Complication of Long-term Alcohol Abuse

**DOI:** 10.15190/d.2023.7

**Published:** 2023-06-30

**Authors:** Emad Singer, Kinal Bhatt, Adesh Prashad, Larri Rudman, Islam Gadelmoula, George Michel

**Affiliations:** ^1^UT MD Anderson Cancer Center, Houston, TX; ^2^Larkin Community Hospital, South Miami, FL

**Keywords:** Marchiafava-Bignami, Wernicke encephalopathy, Alcohol abuse, Neuroimaging, Thiamine, Corticosteroids

## Abstract

Marchiafava Bignami disease (MBD) is a neurological disorder characterized by myelin degeneration and tissue necrosis within the central nervous system. This condition predominantly afflicts individuals with chronic alcohol abuse and malnutrition. The most distinctive pathological feature of MBD is the necrotic degeneration specifically observed in the corpus callosum; however, emerging evidence also indicates the potential involvement of other brain regions. The main pathophysiological mechanisms involve alcohol consumption, which leads to thiamine depletion and disrupts various metabolic pathways. This, in turn, hinders myelin synthesis and impairs signal transmission, resulting in a wide range of symptoms and signs. MBD can manifest in different stages, including acute, subacute, and chronic, each with varying severity. Diagnosing MBD can be challenging due to its presenting symptoms being nonspecific. In the era preceding the development of sophisticated imaging methodologies, the diagnosis of MBD was primarily established through postmortem examination conducted during autopsies. However, with a detailed medical history and imaging modalities such as magnetic resonance imaging (MRI) and computed tomography (CT), it is now possible to diagnose MBD and differentiate it from other diseases with similar clinical presentations. MRI is considered the gold standard for visualizing lesions in the corpus callosum and other affected areas. Also, positron emission tomography (PET), single photon emission computed tomography (SPECT), and magnetic resonance spectroscopy (MRS) could show brain damage in the corpus callosum associated with MBD. MRI-diffusion-weighted imaging (DWI) detects early lesions, while diffusion tensor imaging (DTI) investigates clinical manifestations and recovery. Poor prognostic indicators for MBD include extensive cerebral cortex involvement and severe disturbances in consciousness. Differential diagnosis involves ruling out other alcohol-related disorders, such as neoplastic conditions, Wernicke's encephalopathy, and multiple sclerosis, among others, through careful evaluation. The therapeutic strategies for the management of MBD are currently lacking definitive establishment; however, available evidence indicates that targeted interventions have the potential to induce amelioration. Corticosteroids offer prospective advantages in addressing brain edema, demyelination, and inflammation; research findings present a heterogeneous outcome pattern. Notably, thiamine treatment reduces the likelihood of unfavorable consequences, particularly when administered promptly, and thus is endorsed as the primary therapeutic approach for MBD. This review will highlight this rare disease that many healthcare providers might not be familiar with. By understanding its clinical presentation, differential diagnosis, imaging, and management, medical providers might better identify and diagnose MBD. Raising awareness about this condition can lead to better prevention, early detection, and timely intervention.

## Introduction

Marchiafava-Bignami disease (MBD) is a rare condition characterized by the prominent features of demyelination and necrosis in the corpus callosum and the surrounding subcortical white matter. Individuals with a history of alcoholism and malnutrition represent the most commonly affected population associated with MBD^1^. Italian doctors Ettore Marchiafava and Amico Bignami made the disease's initial discovery in 1903. They noted instances of alcoholic males who went into comatose states and had convulsions right before they passed away. Corpus callosum necrosis was discovered during postmortem tests1. However, MBD has been observed in non-alcoholic patients, suggesting that alcohol consumption may not be the only factor contributing to the development of these degenerative pathologies^2^.

MBD can manifest as an acute, subacute, or chronic condition. Clinically affected individuals display symptoms such as dementia, dysarthria, spasticity, and impaired motor function. Coma or long-lasting dementia may also occur, with some cases exhibiting spontaneous recovery while others prove fatal^3,4^. Imaging techniques such as computed tomography (CT) and magnetic resonance imaging (MRI) aid in visualizing the characteristic lesions. CT scans may reveal hypodense regions within the corpus callosum, while MRI scans typically demonstrate a decrease in T1 signal and an increase in T2 signal within the affected areas. Survivors of MBD may experience interhemispheric disconnection syndrome^5-7^. Furthermore, emerging evidence indicates that MBD can extend beyond the corpus callosum, affecting additional brain regions, including the basal ganglia, cerebral lobes, subcortical areas, and white matter within the hemisphere^8^. When these supplementary brain regions are involved, MBD gives rise to significant neurological dysfunction and carries an unfavorable prognosis.

Postmortem examinations and MRI neuroimaging studies have been conducted on alcoholic patients who do not exhibit liver disease, amnesia, or cognitive dysfunction. These investigations have demonstrated a notable thinning of the corpus callosum, suggesting that alcohol consumption or malnutrition can frequently result in structural damage to this specific brain region, even without the characteristic necrotic lesion associated with MBD^6-8^. This review aims to comprehensively examine the available evidence concerning the etiology, pathophysiology, various presentations, and management of MBD.

## Etiology

Although its specific cause is still unknown, MBD is thought to be caused by a combination of B-complex vitamin deficiency and alcohol-induced neurotoxicity^9^. Other potential causes of MBD include: 1) As a side effect of ketoacidosis brought on by diabetes mellitus or alcoholism, sudden changes in serum osmolality, also known as callosal myelinolysis, can occur^10^; 2) Non-alcoholic malnourishment following bypass surgeries^11^; 3) Additionally, sepsis, carbon monoxide overdose, cerebral malaria, and sickle cell disease have all been related to MBD^12-14^. These variables have been suggested as potential initiators of MBD, demonstrating the disease's complexity and multifaceted character.

## Epidemiology

MBD is predominantly observed in individuals with chronic alcohol use disorder and malnutrition^15^. However, there have been reported cases of MBD in patients who didn't use alcohol but they had diabetes mellites^12,16^. MBD does not exhibit any specific predilection towards a particular race, ethnicity, or geographic region. However, it has a higher incidence in men, likely due to the closer association of the disease with alcohol consumption in males than females^17,18^. The average age of onset of MBD is around 45 years^19^. MBD is considered a very rare condition. A study conducted in the United States reported the existence of 250 published cases of MBD before the year 2001. This finding suggests that many MBD cases might have gone undiagnosed during that period^20^. Similar trends are observed internationally, and it should be noted that the prevalence is underestimated due to the lack of autopsies in all cases^20^.

## Pathophysiology

**Figure 1:**
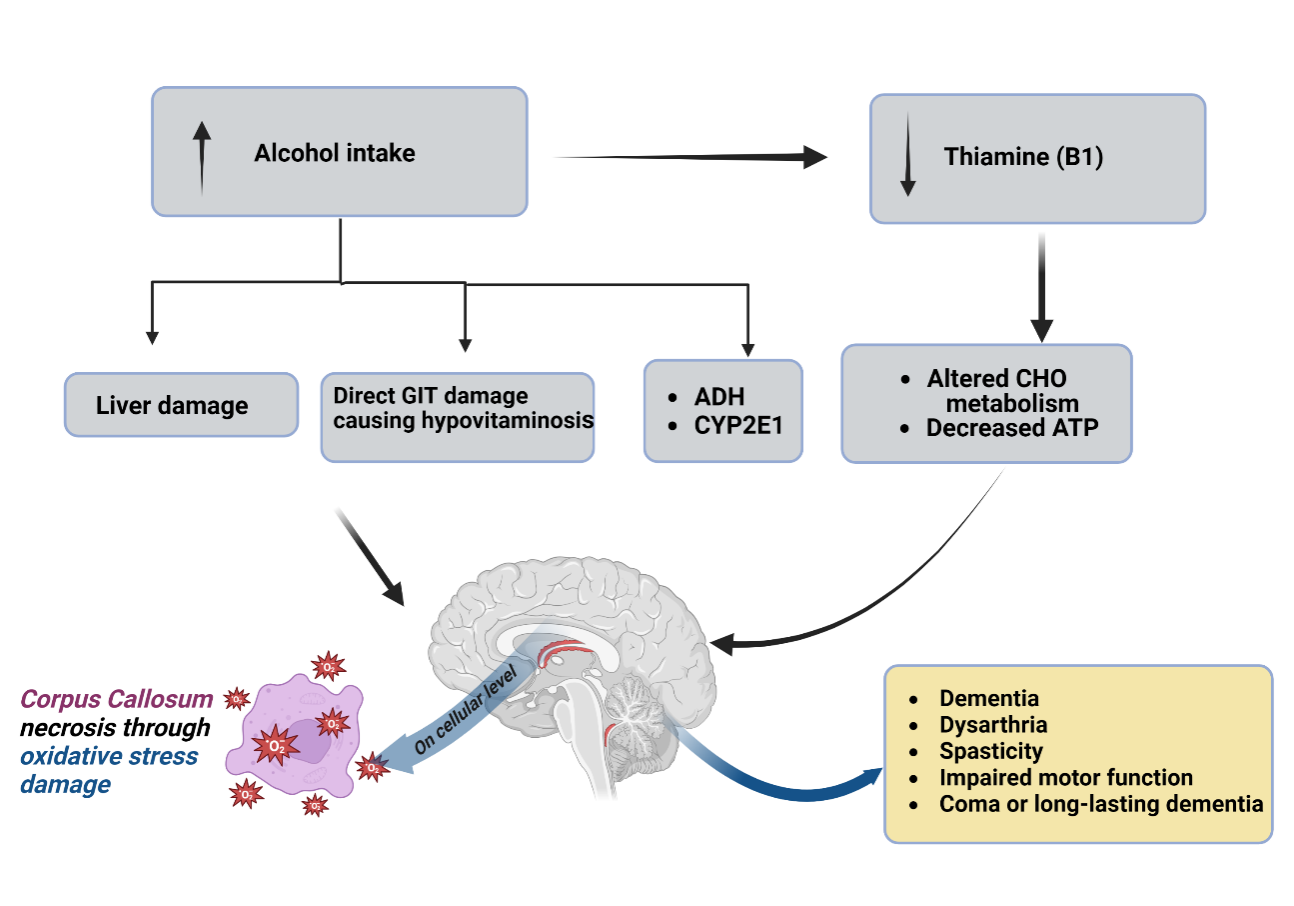
Illustration for proposed mechanism in MBD (created using Biorender.com)

The exact pathophysiology of MBD remains unclear, but several explanations have been proposed (Figure 1):

1) Alcohol, a significant risk factor for many brain diseases, can harm the corpus callosum directly through a number of methods. Direct effects on the gastrointestinal tract can bring on alcohol-related hypovitaminosis, metabolic disturbances brought on by liver damage, decreased reabsorption by renal tubular cells, abnormal lipid metabolism, increased protein catabolism, or dietary deficiencies. Vitamin B1 (thiamine) deficiency is one such condition. Additionally, the oxidative stress that ethanol causes can harm the corpus callosum. These elements can cause atrophy by breaking down the blood-brain barrier, cytotoxic edema, and localized demyelination and necrosis^9,21^.

2) Alcohol can impact the production of proteins that connect cytoskeletal elements in the white matter to cytoskeletal proteins, impede neuronal plasticity, disturb lipid metabolism, and alter neurotransmitter activity^22^. The Aldehyde dehydrogenase (ADH) route is one oxidative mechanism where alcohol can be processed within the central nervous system, leading to oxidative stress. A further mechanism regulated by CYP2E1 may result in elevated levels of acetaldehyde and reactive oxygen species, exacerbating oxidative stress and brain damage^23,24^.

3) Thiamine can impair neurological function by altering the metabolism of carbohydrates and decreasing the amount of adenosine triphosphate accessible (ATP). Due to the enhanced activation of catecholamine neurotransmitters like dopamine brought on by this restriction of ATP, symptoms including delirium, hallucinations, and delusions may result. The production of other neurotransmitters, including acetylcholine and glutamate, is similarly compromised by thiamine shortage, which may be related to pyruvate dehydrogenase malfunction (PHD) malfunction. This malfunction may impair myelin and glutathione formation, subsequently affecting neuronal signaling and the body's capacity to protect itself from oxidative stress^25,26^.

Due to the high myelin concentration of the corpus callosum and its critical function as the main white matter commissure bridging the two hemispheres, the corpus callosum is particularly vulnerable to injury^4^.

## Histopathology

Since diagnostic biopsies in living patients are uncommon and accidental findings, postmortem autopsies are the primary method used to diagnose MBD histologically. The corpus callosum can exhibit necrotizing or cystic lesions, particularly in the genu and body regions. When examined under a microscope, white matter necrosis, a profusion of macrophages (with little inflammatory reaction), infiltration of foamy histiocytes that are positive for CD68 and CD163, small perivascular lymphocytes that are positive for CD3 and CD20, gliosis, and pronounced demyelination are typically seen (with relative preservation of the axons). The middle of the semiovale may be affected symmetrically by this demyelination. Additionally, there is a decrease in oligodendrocyte quantity^27,28^. Other structural regions, including the anterior and posterior commissures, optic chiasm, middle cerebral peduncles, cortex, and brachium pontis, may also be affected by demyelination. Cortical lesions can cause frontal lobe disorders and dementia and are thought to be secondary to callosal injury^27,28^

## Remarks on History and Physical Examination

Patients with specific neurological symptoms with a lengthy history of alcohol abuse and/or malnutrition should be assessed for MBD. Although the clinical

signs of MBD can differ greatly and may not be distinct, some characteristics are frequently seen as follows^29-32^:

1. *Acute Presentation*: Symptoms include loss of consciousness, convulsions, apathy, aggression, disorientation, and psychosis suddenly appearing in this type.

2. *Subacute Presentation*: Patients may exhibit depressive, ataxic, apraxia, agraphia, anomia, dysarthria, and visual symptoms. These signs and symptoms may be a manifestation of the interhemispheric disconnection syndrome, which frequently presents unilaterally.

3. *Chronic Presentation*: This variety includes behavioral problems, auditory delusions, visual hallucinations, and severe global dementia that is progressing. There may also be interhemispheric disconnection syndrome symptoms. Along with the clinical categorization, MBD can be divided into groups according to the clinical status and the degree of brain damage revealed by an MRI:

1. *Type A:* This type is characterized by a more severe clinical manifestation, including a major impairment in consciousness, seizures, hemiparesis, and dysarthria. The corpus callosum is hyperintense and swollen on MRI.

2. *Type B*: This type is characterized by milder symptoms with less impairment of consciousness, such as gait abnormalities, dysarthria, and symptoms of interhemispheric disconnection. Corpus callosum partial lesions are seen on MRI. The prognosis for Type A MBD is often worse, whereas Type B MBD has a somewhat better prognosis^28^.

## Clinical Assessment of MBD

A thorough evaluation of Marchiafava-Bignami disease (MBD) is required, involving collecting a patient's medical and personal history, a physical examination, a neurological and cognitive assessment, blood testing, and imaging scans. The evaluation process consists of the following elements^29^:

1. *Neurological Function Assessment*: To evaluate the overall neurological disability, the modified Rankin Scale (mRS) and modified Oxford Handicap Scale (MOHS) can be utilized. - The Glasgow Coma Scale (GCS) is used to gauge how severely consciousness has been affected.

2. *Assessment of Cognitive Function*: The Mini-Mental State Examination (MMSE), the Abbreviated Mental Test (AMT), and the Montreal Cognitive Assessment (MoCA) can be used to measure cognitive function.

3. *Evaluation of Alcohol Use*: Michigan Alcoholism Screening Test (MAST-C) can be used to evaluate patterns of alcohol consumption and spot alcohol use disorders.

4. *Laboratory tests*: Serum electrolytes are analyzed to rule out electrolyte changes that might result in seizures, altered consciousness, and coma.

- To evaluate liver function, serum transaminases, and bilirubin levels are tested.

- Serum glucose concentrations are assessed to rule out hypo- or hyperglycemia.

- A complete blood count is done to check for macrocytosis and macrocytic anemia and the levels of hemoglobin and platelets.

Toxicology testing may be carried out to find the presence of other chemicals.

- Infectious serology panels for the blood and spinal fluid may be requested.

5. *Imaging Research*: - For imaging examination of MBD, MRI is regarded as the gold standard. It may manifest as symmetrical lesions in the corpus callosum, mainly in the genu, body, or spleen. On an MRI, other lesions in different parts of the brain might also be discernible^30^.

- On CT, the corpus callosum's core section exhibits hypodense lesions^30^.

Additionally, compared to non-alcoholics, MRI results in patients with chronic alcohol use disorder may reveal atrophy and thinning of the corpus callosum as well as decreases in a number of brain indices, including anterior thickness, middle thickness, corpus callosum area, and frontal lobe index. These imaging results might be related to the total amount of alcohol consumed throughout time^15^.

## Advanced imaging modalities for MBD detection

Multiple imaging modalities have been employed to characterize the lesions occurring in the corpus callosum, which are indicative of functional brain damage. These techniques include single photon emission computed tomography (SPECT), magnetic resonance spectroscopy (MRS), and positron emission tomography (PET)^1,31,32^. Studies utilizing [18F]-2-fluoro-2-deoxy-D-glucose PET, technetium99m hexylmethylpropylene aminoexime SPECT, and/or N-isopropyl-p-[123I] iodoamphetamine SPECT have consistently revealed brain hypometabolism and hypoperfusion patterns.

These findings indicate an advanced stage of the disease characterized by permanent brain damage, especially in MBD patients who have experienced poor or partial recovery^1,31,32^. MR perfusion examinations have identified hypoperfusion, except in cases of complete recovery, further confirming the presence of impaired blood flow in individuals with MBD. MR spectroscopy findings have demonstrated an initial increase in choline/creatine, indicating demyelination during the early phase of the disease, followed by a subsequent decrease in N-acetylaspartate/creatine, indicating axonal damage. However, it is important to note that while these findings contribute to our understanding of the disease process, they do not offer a definitive explanation for the etiology of MBD^33-36^. Low apparent diffusion coefficients have been found in studies using MRI diffusion-weighted imaging (DWI), which is a sign of cytotoxic edema in the corpus callosum. Compared to fluid-attenuated inversion recovery, DWI enables the earliest diagnosis of lesions and can spot more widespread callosal lesions in MBD^37-39^. Cytotoxic edema has occasionally been seen in conjunction with other brain regions and may occur before the onset of callosal necrosis, thereby portending a disastrous result. But reports of complete recovery after thiamine therapy have also been made, indicating this is not always the case^37-39^. For examining the clinical signs of MBD and the healing process, diffusion tensor imaging (DTI) shows promise^40^. The aforementioned imaging modalities are summarized in Table 1**.**

**Table 1:** Imaging modalities used for diagnosis of MBD

Imaging Modality	Findings	Clinical Significance
MRI	- Symmetrical lesions in the corpus callosum, mainly in the genu, body, or spleen	- Gold standard for imaging examination of MBD
	- Other lesions in different parts of the brain might also be discernible on MRI	
	- Atrophy and thinning of the corpus callosum	- Associated with chronic alcohol use disorder
	- Decreases in brain indices, including anterior thickness, middle thickness, corpus callosum area, and frontal lobe index	
CT	- Hypodense lesions in the core section of the corpus callosum	- Aid in MBD diagnosis and differentiate it from other causes
SPECT	- Brain hypometabolism and hypoperfusion patterns	- Indicates advanced stage of MBD and permanent brain damage
MRS	- Initial increase in choline/creatine indicating demyelination	- Followed by a subsequent decrease in N-acetylaspartate/creatine
		- Axonal damage
PET	- Brain hypometabolism	
MR Perfusion	- Hypoperfusion	- Confirms impaired blood flow in individuals with MBD
DWI (MRI)	- Low apparent diffusion coefficients indicating cytotoxic edema in the corpus callosum	- Early diagnosis of lesions, more widespread callosal lesions
		- Cytotoxic edema may occur before callosal necrosis
		- Complete recovery possible after thiamine therapy
DTI	- Shows promise for examining clinical signs of MBD and the healing process	

## Management of MBD

Untill now, MBD is not fully understood, and there are no defined treatment protocols. However, management strategies used in Wernicke-Korsakoff syndrome and alcohol use disorder are often employed due to the similarities in etiology and clinical presentation^30,41,42^. Here are some common approaches that have been reported in case reports:

### 1. Thiamine adinistration^43,44^

100 to 250 mg administered intravenously (IV) or intramuscularly (IM) once daily for 3 to 5 days, then 100 mg administered orally three times daily for 1 to 2 weeks, is the recommended dosage for alcohol withdrawal syndrome. Then 100 mg is taken once a day orally as a maintenance dose.

In MBD or Wernicke encephalopathy:

- *Prophylaxis*: 100 to 250 mg intravenously (IV) or intramuscularly (IM) once daily for 3 to 5 days, then 100 mg orally thrice daily for 1 to 2 weeks. Then, a maintenance dose of 100 mg should be administered orally once daily.

- *Treatment:* 200 to 500 mg intravenously three times a day for 2 to 7 days. If the reaction is satisfactory, increase the dose to 250 mg once a day IV or IM for 3 to 5 days (or until the maximal clinical improvement is reached), then reduce it to 30 mg twice daily or 100 mg three times daily for 1 to 2 weeks—lastly, an oral maintenance dose of 100 mg once each day.

- Vitamin B Complex pills may be administered as needed to supplement other B vitamins.

### 2. Corticosteroids

Some case reports have reported high-dose corticosteroids, although the evidence is limited. The dosing regimens may vary, and treatment should be individualized based on the patient's condition and response.

### 3. Amantadine

Amantadine is a dopaminergic and antiviral medication utilized in some MBD instances, albeit it is unclear how it works in this condition. The typical oral dosage for extrapyramidal symptoms is 100 mg twice daily^45,46^.

### 4. Folic acid

Megaloblastic and macrocytic anemias linked to alcohol use disorder and malnutrition can be treated or prevented by supplementing with folic acid at 1 to 5 mg taken orally once daily. Lines of management for MBD are summarized in Table 2.

**Table 2:** Treatment approach of MBD

Treatment Approach	Dosage and Administration	Clinical Application
Thiamine administration	- Alcohol Withdrawal Syndrome: - Intravenously (IV) or intramuscularly (IM): 100-250 mg once daily for 3-5 days, then orally: 100 mg three times daily for 1-2 weeks - Maintenance dose: 100 mg orally once daily	- Recommended dosage for alcohol withdrawal syndrome
	- MBD or Wernicke encephalopathy: - Prophylaxis: IV or IM: 100-250 mg once daily for 3-5 days, then orally: 100 mg three times daily for 1-2 weeks - Maintenance dose: 100 mg orally once daily - Treatment: IV: 200-500 mg three times a day for 2-7 days, then IV or IM: 250 mg once a day for 3-5 days, then reduce to 30 mg twice daily or 100 mg three times daily for 1-2 weeks, followed by an oral maintenance dose of 100 mg daily.	- Prophylaxis and treatment dosage for MBD or Wernicke encephalopathy
Corticosteroids	Dosage regimen should be individualized based on the patient's condition and response	Limited evidence in MBD treatment
Amantadine	Typical oral dosage for extrapyramidal symptoms: 100 mg twice daily	Utilized in some MBD cases
Folic acid	- Megaloblastic and macrocytic anemias linked to alcohol use disorder and malnutrition: - Oral: 1-5 mg once daily	Treatment and prevention of anemias

## Rationale behind MBD treatments

Corticosteroids possess the inherent capacity to attenuate cerebral edema, inhibit demyelination processes, stabilize the integrity of the blood-brain barrier, and alleviate inflammatory responses. Although several investigations have documented favorable outcomes subsequent to corticosteroid administration, the meticulous analysis conducted by Hillbom et al. revealed no statistically significant overall positive impact^1,47,48^. The administration of both corticosteroids and multivitamins to numerous subjects presented a considerable challenge in determining the primary factor responsible for observed improvements in recovery. Although the study conducted by Hillbom et al. did not report any detrimental effects associated with corticosteroid treatment, the concurrent usage of multivitamins confounds the assessment of the exclusive influence of corticosteroids on patient outcomes^1^.

In their investigation, Hillbom et al. noted a noteworthy inclination towards superior overall outcomes among subjects who underwent thiamine treatment compared to those who did not receive such intervention. Particularly, the early administration of thiamine substantially mitigated the risk of an unfavorable outcome^1^. Nevertheless, several treatment failures were also documented^49-51. ^Given the observed correlation between MBD and the manifestation of Wernicke's disease in approximately 15 to 20% of cases, administering vitamins, particularly thiamine, has emerged as a potential therapeutic avenue worth considering^52^. Consistent with previous literature, a substantial prevalence rate of Wernicke's disease (12.4%) has been documented among individuals diagnosed with MBD^1^.

The precise underlying causes of MBD have yet to be fully elucidated, posing challenges in recommending specific therapeutic approaches. Nonetheless, previous reviews have demonstrated that many individuals attained complete recovery following thiamine therapy^1,53-55^. Some instances of treatment failure may be attributed to hospital admission delays after the onset of symptoms. Notably, superior outcomes were observed in subjects receiving thiamine treatment during the acute phase compared to those treated in the chronic phase. Conversely, the efficacy of corticosteroids and other treatment modalities remains speculative.

Contrary to earlier suggestions, thiamine therapy has exhibited favorable or complete recovery outcomes even in non-alcoholic cases, suggesting its efficacy beyond alcohol-related scenarios^39,56,57^.

Consequently, Hillbom et al. assert that thiamine therapy is warranted and recommended as the primary treatment approach for cases of MBD associated with alcoholism, malnutrition, or prolonged vomiting^1^. Additionally, thiamine therapy should be considered in cases where MBD mimics are present, and their thiamine levels fall below the normal range.

## Differential diagnosis

Based on the clinical presentation and the neuroimaging results, there are some possible differential diagnoses for MBD to consider (Table 3). A complete evaluation that includes history taking, physical examination, laboratory tests, and imaging studies is essential for an accurate diagnosis and suitable care. The causes which could mimic MBD^58-61^ are listed in Table 3.

**Table 3:** Treatment approach of MBD

Nutritional deficiencies	Wernicke encephalopathy (Thiamine deficiency)
	Vitamin B12 deficiency
	Folate deficiency
Drugs	Benzodiazepines
	Opioids
	Sedatives
Toxins	Heroin and hallucinogens
	Hydrogen sulfate
	Cyanide
	Methanol
	Carbon monoxide
Metabolic disorders	Endocrine issues, including pituitary and adrenal gland dysfunction, parathyroid and thyroid hormone abnormalities, and others
	Electroylyte disturbance (Sodium, calcium, magnesium, phosphate)
	Hypoxemia and hypercarbia
	Glycemic adjustments
	Osmolarity variations
	Wilson’s disease
	Porphyria
Disorders of the central nervous system	Seizure caused by epilepsy
	Brain injury
	Hypertensive brain disease
	Dementia and psychosis
	Multiple sclerosis
	Central myelinolysis
	Localized tumors
Systemic conditions	Heart diseases
	Blood conditions such as polycythemia, leukemic blast cell crisis, and thrombocytosis.
	Acute or persistent liver failure (especially hepatic encephalopathy).
	Renal failure, both acute and chronic.
	Severe or persistent respiratory failure.
Other conditions	Sepsis
	Delirium
	Encephalitis
	Meningitis

## How to differentiate between MBD and MBD mimics

Firstly, previous research has primarily identified MBD in individuals who suffer from alcoholism or malnutrition. The association between MBD and dietary deficiency in alcoholics is particularly robust, as excessive alcohol consumption can lead to thiamine depletion even without caloric malnutrition. On the other hand, MBD mimics are typically linked to conditions such as cerebral infection, epilepsy, withdrawal from antiepileptic drugs, hypoglycemia, high altitude sickness, and systemic lupus erythematosus^1,14,62^.

Secondly, the clinical manifestations displayed by individuals diagnosed with MBD distinguish them from those observed in conditions resembling MBD. MBD patients frequently exhibit severe symptoms encompassing alterations in cognitive function, impaired motor coordination, memory impairment, speech difficulties, pyramidal tract signs, and prolonged disorientation persisting for several weeks. In contrast, mimicking disorders typically present with milder symptoms resolved within a week. Notably, delirium, impaired consciousness, and seizures are commonly observed in both MBD and MBD mimics; however, seizures appear to be more frequent in conditions mimicking MBD^1,14,63,64^.

MBD does not manifest with prototypical clinical presentations. Although split-brain syndrome was previously recognized as a distinguishing feature, its diagnostic significance has diminished in light of the advancements in contemporary brain imaging methods that can identify callosal lesions. This allows for promptly diagnosing confused patients in emergency room settings^65^. Signs of interhemispheric disconnection may be challenging to identify without neuropsychological testing, especially in individuals with reduced levels of consciousness. In chronic MBD cases, a range of disconnection signs may be observed depending on the location of lesions within the corpus callosum. While disconnection represents a typical clinical feature of the disease, it can easily go unnoticed if not actively sought out, and it may even be present in mimicking conditions^1,14,63-65^.

Thirdly, the lesions in the corpus callosum exhibit different locations and distributions between MBD and MBD mimics. In MBD mimics, lesions are commonly found in splenium, whereas only one-third of MBD subjects exhibit single splenial lesions. Notably, lesions in MBD can be found in various regions of the corpus callosum or may be distributed throughout the entire structure. The classic presentation of large lesions symmetrically located in the midline of the splenium is no longer the sole characteristic lesion pattern associated with MBD. It has been observed that certain MBD lesions exhibit enhancement with gadolinium, although no such lesion enhancement has been reported in conditions that mimic MBD^1^.

## Prognosis

Patients with MBD may have very different prognoses. While some may enjoy a full recovery, others may endure years with unabated symptoms. But in extreme circumstances, the illness can cause death via the comatose condition. There is a theory that the size of the lesions in the corpus callosum may impact the prognosis. Compared to lesions that extend into the convolution white matter, incomplete lesions that spare the superior commissure fibers have a better prognosis. A worse prognosis and a higher chance of severe dementia are also linked to extracallosal lesions, cerebral lobe dysfunction, significant consciousness disturbance, and heavy alcohol usage. Early diagnosis and fast application of good treatment are essential for a patient to recover. Serial MRI scans have shown instances in which prompt diagnosis and appropriate care resulted in the complete disappearance of corpus callosum abnormalities^66^.

## Conclusion

MBD is a rare neurological disorder that predominantly affects individuals with chronic alcohol use and malnutrition. The distinguishing feature of MBD is the presence of necrotic changes in the corpus callosum, although involvement of other brain regions can also occur. MBD is diagnosed through detailed medical history and imaging techniques such as MRI, CT, PET, SPECT, and MRS. Prognosis is influenced by various factors, with poor indicators including extensive cerebral cortex involvement and severe disturbances in consciousness. Differential diagnosis involves ruling out other alcohol-related disorders and neoplastic conditions. While therapeutic strategies lack definitive establishment, thiamine treatment is recommended as the primary therapeutic approach, promptly administered to reduce unfavorable consequences. The efficacy of combined therapy with thiamine and corticosteroids remains uncertain due to the condition's rarity. Further research is needed to evaluate the effectiveness of interventions in MBD management.
